# Intralenticular Metallic Foreign Body After Pediatric Ocular Trauma

**DOI:** 10.3390/diagnostics16121889

**Published:** 2026-06-17

**Authors:** Bogumiła Wójcik-Niklewska, Adriana Błaszczyk-Windak, Martyna Marcoll, Anna Kamińska, Dorota Wyględowska-Promieńska

**Affiliations:** 1 Department of Pediatric Ophthalmology, Faculty of Medical Sciences in Katowice, Medical University of Silesia, 40-514 Katowice, Poland; 2 Department of Pediatric Ophthalmology, Professor Kornel Gibiński University Clinical Center, Medical University of Silesia, 40-514 Katowice, Poland; 3 Students Scientific Society, Department of Ophthalmology, Faculty of Medical Sciences in Katowice, Medical University of Silesia, 40-514 Katowice, Poland; s80758@365.sum.edu.pl (A.B.-W.); marcollmartyna@gmail.com (M.M.); aniakam554@wp.pl (A.K.); 4 Department of Ophthalmology, Faculty of Medical Sciences in Katowice, Medical University of Silesia, 40-514 Katowice, Poland; 5 Department of Ophthalmology, Professor Kornel Gibiński University Clinical Center, Medical University of Silesia, 40-514 Katowice, Poland

**Keywords:** eye foreign bodies, corneal injuries, child, lens implantation, intraocular

## Abstract

Intraocular foreign bodies penetrating the eye can lead to serious complications, including endophthalmitis, and therefore require urgent removal. We present the case of a 9-year-old boy with an intraocular foreign body lodged in the lens with a corneal flap wound. The injury occurred while hammering a bicycle frame. The patient presented with sudden pain, tearing, and decreased visual acuity in the left eye. On admission, the left eye distance best-corrected visual acuity (BCVA) was 0.4 and intraocular pressure (IOP) was 17 mmHg. Slit-lamp examination of the left eye revealed a full-thickness corneal flap wound, a traumatic cataract, and a foreign body located centrally within the lens. B-scan ultrasonography demonstrated an echogenic focus within the lens consistent with an intralenticular metallic foreign body, with a normal posterior segment, a regular appearance of the optic disc, and an attached retina. The patient underwent phacoaspiration of the traumatic cataract with intraocular lens implantation and simultaneous removal of the foreign body. Given the corneal flap wound located in the visual axis and the absence of ocular hypotony, the decision was made not to place a corneal suture. At discharge, BCVA improved to 1.0, with IOP of 17 mmHg and normal fundus appearance.

**Figure 1:**
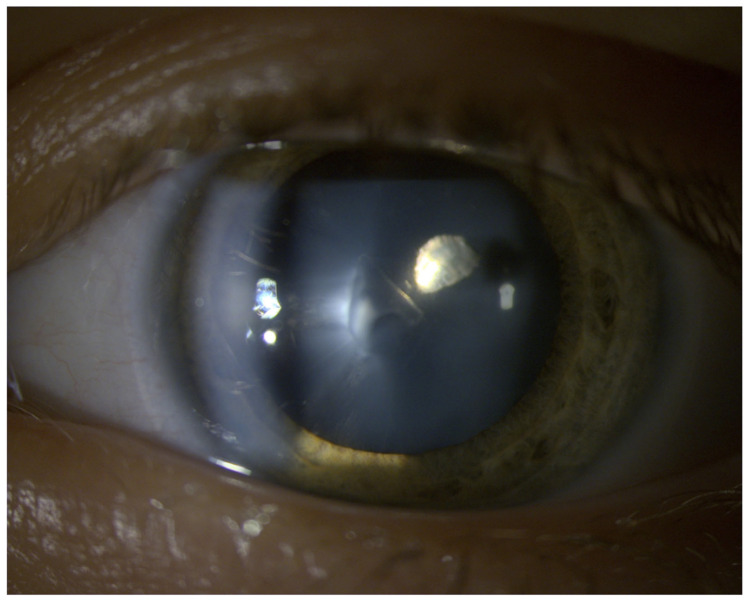
We present the case of a 9-year-old boy with an intraocular foreign body lodged in the lens with a corneal flap wound. The trauma occurred while hammering a bicycle frame—a mechanism generating small, high-velocity fragments. Intralenticular foreign bodies constitute a relatively uncommon subset of intraocular foreign bodies, accounting for approximately 2% of all intraocular foreign bodies. They may present with limited initial symptoms despite significant structural damage [1]. According to the classification and prognostic systems, such high-kinetic-energy impacts often result in complex penetrating injuries that require a structured approach to trauma management [2]. The patient presented with sudden pain, tearing, and decreased visual acuity in the left eye. Upon admission, clinical examination of the left eye disclosed distance best-corrected visual acuity (BCVA) of 0.4 and intraocular pressure (IOP) of 17 mmHg. Slit-lamp examination of the left eye prior to surgical treatment demonstrated a metallic foreign body located in the central part of the lens (Figure 1). Prior to the transfer to our facility, a computed tomography (CT) scan was performed at the referring centre, which confirmed the presence of the metallic foreign body and ruled out additional posterior fragments. To assess the integrity of the posterior segment upon admission, B-scan ultrasonography was performed. It revealed an echogenic focus within the lens consistent with an intralenticular metallic foreign body, with a normal posterior segment, a regular appearance of the optic disc, and an attached retina. These findings correlate with a significant reduction in visual acuity and represent a typical clinical presentation observed in penetrating open-globe injuries caused by a high-velocity foreign body. Favourable visual outcomes can be achieved with timely surgical intervention when the posterior segment remains unaffected [3,4]. The patient underwent phacoaspiration of the traumatic cataract with intraocular lens implantation and simultaneous removal of the foreign body.

**Figure 2:**
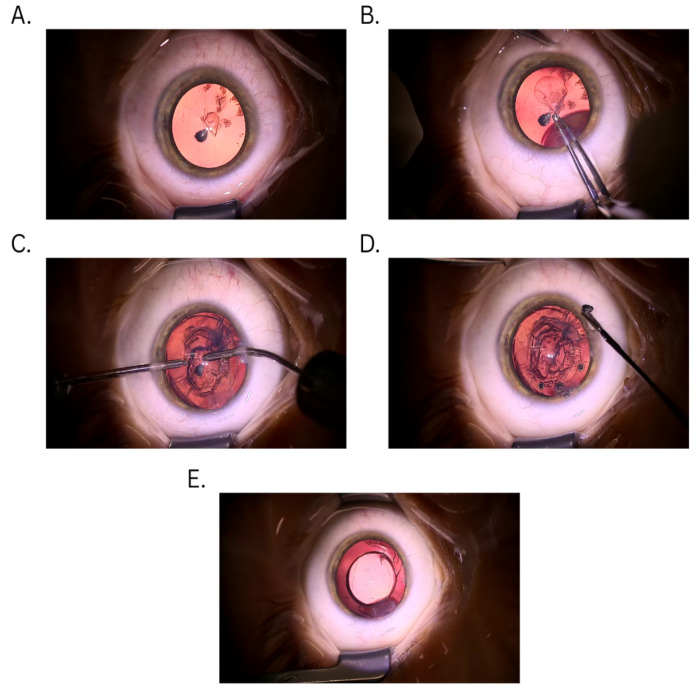
The figure provides the sequence of intraoperative images documenting the surgical treatment of a penetrating injury to the left globe with partial traumatic cataract and an intralenticular metallic foreign body. Panel (**A**) illustrates the opacified lens; traumatic cataract and irregular damage to the anterior lens capsule is visible. Following anterior capsulorhexis (**B**) and aspiration of lens material (**C**), the foreign body is clearly visible and stabilized using surgical instruments. The metallic foreign body is removed from within the lens using microsurgical instruments (**D**), followed by cleansing of the capsular bag. After confirmation of lens capsule stability, an intraocular lens was implanted (**E**). Given the corneal flap wound located in the visual axis and the absence of ocular hypotony, the decision was made not to place a corneal suture. Prophylactic antibiotic therapy was administered using topical levofloxacin to mitigate the risk of infection. At discharge, BCVA improved to 1.0, with IOP of 17 mmHg and normal fundus appearance. From a management perspective, the composition of the intralenticular foreign body is a critical determinant of the surgical timeline. While inert materials such as glass or high-grade plastics may occasionally be monitored if the lens capsule seals spontaneously and the lens remains clear, metallic fragments are notoriously reactive. Metallic intralenticular foreign bodies almost universally necessitate removal to prevent progressive cataract and the toxic effects of siderosis [3]. In the pediatric population, the resulting traumatic cataract carries the added risk of deprivation amblyopia, which can occur if a dense cataract is left untreated during critical periods of visual development [2,5]. The decision to perform primary intraocular lens implantation depends on the integrity of the capsular bag and intraoperative stability, both of which are key determinants of postoperative visual rehabilitation [6]. However, it is crucial to emphasize that such a decision must be evaluated strictly on a case-by-case basis. The occurrence of postoperative infection (endophthalmitis) is highly unpredictable; the conventional and often more prudent approach involves a two-stage procedure. While our single-stage approach yielded a favourable outcome, supported by a protocol of prophylactic antibiotics (such as topical levofloxacin/amikacin and, when indicated, systemic antibiotics), the inherent risks of severe intraocular infection must be carefully weighted by the readers. In contrast to posterior segment injuries, which carry a guarded prognosis due to the risks of retinal detachment and endophthalmitis, isolated intralenticular injuries offer an excellent visual prognosis when addressed with a controlled, modern surgical technique. This case highlights that a structured, timely surgical approach in a 9-year-old can result in full visual recovery (BCVA 1.0), emphasizing the importance of rapid pediatric ophthalmic trauma care and the need for surveillance to identify posterior segment damage that may manifest following high-velocity impacts [7].

## Data Availability

The original contributions presented in this study are included in the article. Further inquiries can be directed to the corresponding author.

## Abbreviations

The following abbreviations are used in this manuscript:

BCVA	best-corrected visual acuity
IOP	intraocular pressure

## References

[1] Greven C.M., Engelbrecht N.E., Slusher M.M., Nagy S.S. (2000). Intraocular foreign bodies: Management, prognostic factors, and visual outcomes. Ophthalmology.

[2] Mester V., Kuhn F. (2002). Intraocular foreign bodies. Ophthalmol. Clin. N. Am..

[3] Loporchio D., Mukkamala L., Gorukanti K., Zarbin M., Langer P., Bhagat N. (2016). Intraocular foreign bodies: A review. Surv. Ophthalmol..

[4] Thompson J.T., Parver L.M., Enger C.L., Mieler W.F., Liggett P.E., National Eye Trauma System (1993). Infectious endophthalmitis after penetrating injuries with retained intraocular foreign bodies. Ophthalmology.

[5] Arora R., Sanga L., Kumar M., Taneja M. (2000). Intralenticular foreign bodies: Report of eight cases and review of management. Indian J. Ophthalmol..

[6] Han S., Wang T., Jia J., Sun S., Fan Y., Yang G., Yang Z. (2019). Visual Outcomes and Prognostic Factors of Intralenticular Foreign Bodies in a Tertiary Hospital in North China. J. Ophthalmol..

[7] Sen P., Shah C., Sen A., Jain E., Mohan A. (2018). Primary versus secondary intraocular lens implantation in traumatic cataract after open-globe injury in pediatric patients. J. Cataract. Refract. Surg..

